# Rice Varieties Intercropping Induced Soil Metabolic and Microbial Recruiting to Enhance the Rice Blast (*Magnaporthe Oryzae*) Resistance

**DOI:** 10.3390/metabo14090507

**Published:** 2024-09-20

**Authors:** Xiao-Qiao Zhu, Mei Li, Rong-Ping Li, Wen-Qiang Tang, Yun-Yue Wang, Xiao Fei, Ping He, Guang-Yu Han

**Affiliations:** State Key Laboratory for Conservation and Utilization of Bio-Resources in Yunnan, Ministry of Education Key Laboratory of Agriculture Biodiversity for Plant Disease Management, College of Plant Protection, Yunnan Agricultural University, Kunming 650500, China; zhuxiaoqiao111@gmail.com (X.-Q.Z.); limei000111@gmail.com (M.L.); lirongping001@gmail.com (R.-P.L.); tangwenqiang1015@gmail.com (W.-Q.T.); yunyuewang@ynau.edu.cn (Y.-Y.W.); feixiao000111@gmail.com (X.F.)

**Keywords:** rice intercropping, rice blast resistance, soil metabolome, bacterial microbiome, control efficiency

## Abstract

[Background] Intercropping is considered an effective approach to defending rice disease. [Objectives/Methods] This study aimed to explore the resistance mechanism of rice intraspecific intercropping by investigating soil metabolites and their regulation on the rhizosphere soil microbial community using metabolomic and microbiome analyses. [Results] The results showed that the panicle blast disease occurrence of the resistant variety Shanyou63 (SY63) and the susceptible variety Huangkenuo (HKN) were both decreased in the intercropping compared to monoculture. Notably, HKN in the intercropping system exhibited significantly decreased disease incidence and increased disease resistance-related enzyme protease activity. KEGG annotation from soil metabolomics analysis revealed that phenylalanine metabolic pathway, phenylalanine, tyrosine, and tryptophan biosynthesis pathway, and fructose and mannose metabolic pathway were the key pathways related to rice disease resistance. Soil microbiome analysis indicated that the bacterial genera *Nocardioides*, *Marmoricola*, *Luedemannella*, and *Desulfomonile* were significantly enriched in HKN after intercropping, while SY63 experienced a substantial accumulation of *Ruminiclostridium* and *Cellulomonas.* Omics-based correlation analysis highlighted that the community assembly of *Cellulomonas* and *Desulfomonile* significantly affected the content of the metabolites D-sorbitol, D-mannitol, quinic acid, which further proved that quinic acid had a significantly inhibitory effect on the mycelium growth of *Magnaporthe oryzae*, and these three metabolites had a significant blast control effect. The optimal rice blast-control efficiency on HKN was 51.72%, and Lijiangxintuanheigu (LTH) was 64.57%. [Conclusions] These findings provide a theoretical basis for rice varieties intercropping and sustainable rice production, emphasizing the novelty of the study in elucidating the underlying mechanisms of intercropping-mediated disease resistance.

## 1. Introduction

Rice (*Oryza sativa* L.), an important food crop, is widely grown all over the world [1]. Steady rice production is the guarantee for global food security and development [2]. Rice blast, caused by the fungus *M. oryzae* (anamorph: *Pyricularia oryzae*), is a highly prevalent and devastating disease that can lead to yield losses of up to 70–80% [3]. With the global expansion of sustainable agriculture, people have started looking into new and more potent green environmental protection strategies to control rice blast disease [4].

The utilization of intraspecific diversity for planting is a promising approach for improving agricultural sustainability by increasing biodiversity in cultivated systems. Rice variety mixing (intercropping) can reduce the blast severity of sensitive varieties by 94% on average [5]. Fau et al. found that mixed planting of different varieties of potato could reduce the severity of potato late blight in susceptible varieties [6]. Danish winter wheat variety testing trials revealed that variety combinations effectively reduced the occurrence of *Septoria tritici blotch* [7]. In particular, such systems may be able to reduce the use of pesticides and naturally achieve the effects of disease resistance, stable yield, and sustainable production. Intercropping has been proven to be an effective strategy for preventing and controlling the outbreak of rice blast [5,8]. Many hypotheses have been put forward about the mechanism of controlling rice blast by using rice diversity and mixed intercropping, including: (1) the dilution effect of pathogens. Van Bruggen reported that planting disease-resistant varieties per unit area can reduce the density of susceptible varieties and the number of pathogenic bacteria [9], (2) physical barrier effect. Ratnadass revealed that resistant rice varieties can act as a physical barrier to limit the spread of pathogens [10], (3) micro-ecological effect. Qin found that by improving the canopy temperature, humidity, ventilation, and light of rice plants, a field microclimate unfavorable to the occurrence of diseases could be formed [11], (4) induced resistance. Zhu has shown that pathogens that are non-toxic to host genotypes may develop effective resistant responses to toxic hosts [12], and (5) nutritional physiology. Ning found that rice mixed sowing can enhance the disease resistance of rice by improving the efficiency of nutrient absorption [13]. Intercropping has garnered significant attention due to its potential to increase crop productivity and ecosystem services. Notably, it has been proven to increase the genetic diversity of rice plants, which leads to modifications in the soil environment and elicits fundamental immune responses to pathogens [8,14]. Recent studies have reported the pivotal role of underground interactions in driving productivity in intercropping systems [15]. Pelissier et al. introduced the concept of NMS (neighbor-modulated susceptibility), which postulates that basic immunity and susceptibility of rice plants to pathogens can be regulated by the presence of neighboring, genetically distinct rice cultivars. However, the intricate mechanisms governing these underground interactions remain elusive [16].

The significance of the rhizosphere soil as a vital region for crop nutrient transformation and soil microbial activity is becoming increasingly apparent [17]. Soil microorganisms regulate soil nutrient absorption and plant development by promoting interspecific and intraspecific interaction [15]. Soil microbial communities with high species diversities exhibit stronger resistance to pathogen invasion [18,19]. Maize-peanut intercropping has been found to enrich the rhizosphere soil with beneficial bacteria’s relative abundance [20]. The intraspecific intercropping of *Radix pseudostellariae* has enriched the diversity of fungal and bacterial communities, resulting in a significant reduction in the relative abundance of pathogenic *Fusarium*, increased the beneficial *Pseudomonas* and *Burkholderia*, and effectively controlled a variety of soilborne diseases caused by continuous monoculture [21]. Furthermore, when compared to monoculture systems, the intercropping of maize with soybean significantly reduced soybean root rot disease caused by *Fusarium* [22].

Intercropping exerts a profound influence on the assembly of rhizosphere microbial by various metabolites it releases [17,23]. The secondary metabolites play a pivotal role in signaling, nutrition uptake, immune system expression, and susceptibility to infections [16,24]. Notably, adjacent cassava plants have been shown to stimulate peanut roots to release ethylene, increasing the presence of actinomycetes and reshaping the composition of rhizosphere microbes [25]. Similarly, the chemical signals released by onion root exudates alter the recruitment of rhizosphere microbiome, thereby improving the adaptability of tomato plants [26]. In the watermelon/rice intercropping system, the soil metabolites of rice recruited a variety of Gram-positive bacteria and actinomycetes in rhizosphere soil and effectively controlled the Fusarium wilt of watermelon [27]. Additionally, benzoxazine compounds secreted from wheat and corn have been found to reassemble rhizosphere fungal and bacterial communities, increasing jasmonic acid signaling and plant defense [28]. Despite these advancements, the underground resistant mechanism of rice intraspecific intercropping and the pathway of how soil metabolic processes affect the rhizosphere microbiome assembly were still unknown.

To address these knowledge gaps, this study aims to investigate the resistant and susceptible rice varieties in monoculture and intercropping planting systems. By utilizing metabolome sequencing (LC-MS/MS) and microbiome sequencing (16 S rRNA high-throughput), we aim to identify significant bacterial communities and functional metabolites in the rice rhizosphere soil of the intercropping system. Furthermore, we will verify the control effect of the significantly correlated metabolites on rice blast through in vitro and in vivo experiments.

## 2. Materials and Methods

### 2.1. Randomized Complete Block Design (RCBD)

On the basis of a past three-year field comparison trial of rice variety combinations, the high-quality rice variety combination (the resistant variety Shanyou63 and the susceptible variety Huangkenuo) was selected according to the disease incidence, genetic differentiation, and plant growth. Field experiments of rice intercropping were performed in Jianshui County, Yunnan, China (23°36′ N, 102°46′ E) in March to October 2022. The experimental design used in this study was a randomized complete block design. Four treatments (planting patterns) were set: (1) intercropping of Huangkenuo (Inter-HKN), (2) intercropping of Shangyou63 (Inter-SY63), (3) monoculture of Shangyou63 (Mono-SY63), and (4) monoculture of Huangkenuo (Mono-HKN), with three subplots per treatment as biological replicates. The subplot area in the experiment was 5.2 m × 2.93 m. For intercropping, 20 columns of Shanyou63 and 5 columns of Huangkenuo were planted on 10 rows per plot. For monoculture, ten columns of Shanyou63 and ten columns of Huangkenuo were planted on ten rows in every plot.

### 2.2. Disease Investigation and Soil Sample Collection

The rice panicle blast occurrence of SY63 and HKN in four planting patterns was investigated at the yellow ripeness stages. Twelve rice plants were selected to conduct the disease investigation; the investigation standard was as follows: (Class 0: healthy panicle and plants; Class 1: 5% panicle loss; Class 2: 5–20% panicle loss; Class 3: 20–50% panicle loss; Class 4: 50–70% panicle loss; Class 5: 70–100% panicle loss [29]. Incidence Rate (IR) = (Number of Diseased Plants/Total Number of Plants Investigated) × 100%. Disease Index (DI) = 100 × Σ [(Number of Plants in Each Disease Class × Representative Value of That Class)/(Total Number of Plants Investigated × Highest Representative Value)].

At the early rice heading stage, the rhizosphere soil samples of Mono-HKN, Mono-SY63, Inter-HKN, and Inter-SY63 were collected, referring to Zhang [30] with minor modifications. Three rice plants were uprooted from the soil and gently shaken to remove the extra soil for one soil sample. Soil from intercropping was separately sampled from two rice varieties. The roots of 2–3 tillers were cut into a centrifuge tube filled with 35 mL of 1× Phosphate Buffer Saline (PBS) solution. The rhizosphere soil samples were collected after centrifugation at 1500× *g* for soil metabolites and rhizosphere bacterial community sequencing. Residual rhizosphere soil is used to determine soil disease-related enzyme activity. Soil enzymes urease (UE), nitrate reductase (NR), and acid protease (Acp) activities were determined by the reductase kit (Nanjing Mofan Biotechnology Co., Ltd., Nanjing, China), and the manipulation was referred to the kit instructions accordingly.

### 2.3. Extraction of Soil Metabolites

Soil metabolites were extracted from soil samples according to the methodology of Li et al. [3]. Briefly, internal standard extractant (20 mg soil, 400 μL 70% methanol) was centrifuged at 1500× *g* for 5 min. The samples were then iced for 15 min, and 300 μL supernatant was collected at −20 °C after 30 min centrifuged at 12,000× *g* and cooled at 4 °C for 10 min. The new supernatant was collected after being centrifuged at 12,000× *g* and 4 °C for 3 min for sequencing. A mass spectrometer (Q-Trap MS-6545, LECO, St. Joseph, MI, USA) and an ultra-high-performance liquid chromatograph (Agilent 7890, Santa Clara, CA, USA) were used to identify soil metabolites at Wuhan Maiteweier Biotechnology Co., Ltd. (Wuhan, China).

### 2.4. Quantification of Soil Metabolites by LC-MS/MS

The metabolites were analyzed by an LC-ESI-MS/MS system (UPLC, ExionLC AD, https://sciex.com.cn/, accessed on 1 June 2023; MS, QTRAP^®^ System, https://sciex.com/, accessed on 20 June 2023). Waters ACQUITY UPLC HSS T3 C18 (1.8 µm, 2.1 mm * 100 mm) was used for the UPLC column at 40 °C. The flow rate was set as 0.4 mL/min; the injection volume was set as 2 µL; and the solvent system was configured as water (0.1% formic acid): acetonitrile (0.1% formic acid); the gradient program was set as 95:5 *v*/*v* at 0 min, 10:90 *v*/*v* at 11.0 min, 10:90 *v*/*v* at 12.0 min, 95:5 *v/v* at 12.1 min, 95:5 *v*/*v* at 14.0 min. The draft data obtained by the LC-MS assay were first converted into the mzML format using ProteoWizard software (3.x version). Peak extraction, alignment, and retention time correction were conducted using the XCMS program. The peaks were filtered when the deletion rate was more than 50%. Metabolic identification information was obtained by scanning the self-built database and combining the public database with met DNA.

### 2.5. Differential Analysis of Soil Metabolites

Principal component analysis (PCA) was performed with R (Version 3.5.0) to find out the effects of different planting patterns on soil metabolites. Differential metabolites between treatments were screened by variable importance projection (VIP) value ≥ 1 and *p* < 0.05. R package MetaboAnalystR (4.0 version) was selected to analyze the orthogonal partial least squares discriminant analysis (OPLS-DA) (including incorporating score plots and permutation plots). VIP values were obtained from the OPLS-DA analysis. A total of 200 permutation tests were performed to filter the overfitted data.

### 2.6. Genomic DNA Extraction and PCR Amplification of Rhizosphere Soil

Total microbial genomic DNA was extracted from rhizosphere soil samples according to the manufacturer’s instructions of the EZNA^®^ soil DNA Kit (Omega Bio-Tek, Norcross, GA, USA). The quality of extracted DNA was determined by agarose gel electrophoresis (1.0%) and a NanoDrop^®^ ND-2000 spectrophotometer (Thermo Scientific Inc., Waltham, MA, USA). The V3-V4 region of the bacterial 16 S rRNA gene was amplified using the primer pairs 338 F (5′-ACTCCTACGGGAGGCAGCAG-3′) and 806 R (5′-GGACTACHVGGGTWTCTAAT-3′) [29]. The PCR product was retrieved from 2% agarose gel, purified by using the AxyPrep DNA Gel Extraction Kit (Axygen Biosciences, Union City, CA, USA), and quantified by using Quantus™ Fluorometer (Promega Corporation, Madison, WI, USA). Purified amplicons were pooled in equimolar amounts and then sequenced on an Illumina NovaSeq PE250 platform (Illumina, San Diego, CA, USA) at Majorbio Bio-Pharm Technology Co., Ltd. (Shanghai, China) for the construction of pair-end reads and library preparations.

### 2.7. Data Optimization of Soil Microbes

Raw FASTQ files were demultiplexed using an in-house Perl script and then quality-filtered by fastp version 0.19.6 [30] and merged by FLASH version 1.2.7 [31] with the following criteria: (i) the 300 bp reads were truncated at any site receiving an average quality score of <20 over a 50 bp sliding window, and the truncated reads shorter than 50 bp were discarded; reads containing ambiguous characters were also discarded; (ii) only overlapping sequences longer than 10 bp were assembled according to their overlapped sequence. The maximum mismatch ratio of the overlap region is 0.2. Reads that could not be assembled were discarded; (iii) Samples were distinguished according to the barcode and primers, and the sequence direction was adjusted, allowing exact barcode matching and 2 nucleotide mismatches in primer matching. Then the optimized sequences were clustered into operational taxonomic units (OTUs) using UPARSE 7.1 [32,33] with a 97% sequence similarity level. The most abundant sequence for each OTU was selected as a representative sequence.

### 2.8. OTU Clustering

Using Uparse (version 7.0.1090 http://drive5.com/uparse/, accessed on 1 November 2023), according to the similarity of 97% to the OTU sequence cluster. In order to obtain the species classification information corresponding to each OTU, the RDPclassifier Bayesian algorithm was used to conduct taxonomic analysis on 97% of OTU representative sequences with similar levels. The phylogenetic relationships and taxonomic analyses of all OTU representative sequences were then compared in Silva (Release138, http://www.arb-silva.de, accessed on 4 November 2023) database.

### 2.9. Differential Analysis of Soil Microbial Communities

Beta diversity of bacterial communities was calculated by principal coordinate analysis (PCoA), following the principle of the Bray-Curtis dissimilarity matrix (Vegan v2.5–3 package). Alpha diversity richness (Sobs index) was used to assess the impact of intercropping on the rhizosphere bacterial population. The species difference analysis determined which bacterial genera were substantially more prevalent across the various groups (*p* < 0.05).

### 2.10. KEGG Annotation and Enrichment Analysis

Kyoto Encyclopedia of Genes and Genomes (KEGG, KEGG compound database, http://www.kegg.jp/kegg/compound/, accessed on 1 December 2023) was used to annotate the identified microbes and metabolites. The annotated microbes and metabolites were then mapped to the KEGG Pathway database (http://www.kegg.jp/kegg/pathway.html, accessed on 1 December 2023) to screen the differential pathways enriched significantly. The hypergeometric test’s *p*-value was used to identify the pathways. Enriched pathway graphs were drawn using GraphPad Prism v6.01 (GraphPad Software Inc., La Jolla, CA, USA).

### 2.11. Correlation and Statistical Analysis

Quantitative data of multiple omics were used for correlation analysis, the correlation coefficients between different substances were calculate, and heat maps were made according to the coefficient results. The multi-omics advanced correlation clustering heat map was drawn according to spearman correlation calculation method. The correlation analysis was conducted on the Metware Cloud platform https://cloud.metware.cn/. The statistical analysis of soil biochemical properties and rice blast occurrence degree were evaluated by ANOVA (Analysis of Variance) for detecting overall differences among the treatments of Mono-HKN, Mono-SY63, Inter-HKN, and Inter-SY63. Tukey’s honestly significant difference (HSD) test was employed as a post-hoc analysis to specifically compare and determine the statistical significance of pairwise differences among these treatments, *p* < 0.05 was regarded as statistically significant. Correlation graphics were drawn using Origin Pro (version 9.0; Origin Laboratory Inc., Northampton, MA, USA).

### 2.12. The Rice Blast Control Effects of Three Significantly Correlated Metabolites

To assess the inhibitory effects of *correlated metabolites* (D-sorbitol, D-mannitol, and quinic acid) on the mycelial growth of *M. oryzae*, we initially conducted the plate confrontation assay. The 8 mm *M. oryzae* mycelial discs obtained with a cork borer were inoculated in the center of the potato dextrose agar (PDA) media, which contained D-sorbitol (concentrations of 50 mM, 100 mM, 200 mM, 400 mM), D-mannitol (concentrations of 50 mM, 100 mM, 200 mM, 400 mM), and quinic acid (concentrations of 0.5 mg/mL, 1 mg/mL, 10 mg/mL, 20 mg/mL). The pathogen was inoculated onto blank PDA medium (without metabolites) as a control treatment. When the growth of *M. oryzae* in the control treatment reached the entire petri dish at 28 °C in the dark, the colony diameters were measured using the cross-method, and the inhibition rates of mycelial growth were calculated, with triplicate repetitions for each treatment. Data analysis and significance testing followed the analytical methods outlined in Section 2.11.

To further clarify the efficacy of these metabolites in controlling rice blast disease, pot-based inoculation experiments were conducted. Rice seeds were germinated in darkness at 28 °C for 2 days (100% humidity), and the germinated seeds were then transplanted into soil. Metabolites-inoculation treatments were applied when the rice grew to the three-leaf-and-one-bud stage. Under sterile conditions, the *M. oryzae* strain GUY11 was cultured at 28 °C for 7 days, and a spore suspension at a concentration of 2 × 10^5^ spores/mL was prepared. Metabolites solutions of D-sorbitol (50 mM, 100 mM, and 200 mM), D-mannitol (50 mM, 100 mM, and 200 mM), and quinic acid (10 mg/mL, 20 mg/mL, and 50 mg/mL) were sprayed onto the rice leaves, respectively, and GUY11 spore suspension was sprayed onto the rice leaves 24 h later. The treatment only sprayed with sterile water was set as control. The inoculated rice plants were transferred into a culture chamber and maintained at a humidity of 100% in darkness for 24 h at 28 °C after inoculation, followed by alternating periods of light (16 h) and darkness (8 h) at constant humidity of 60–80% for 6 days at 28 °C. Disease surveys were conducted 7 days post-inoculation, during which lesion counts and leaf area measurements were recorded to calculate disease incidence and disease severity index. Data analysis and significance testing were conducted using the same analytical methods as described in Section 2.11.

## 3. Results

### 3.1. Rice Intercropping Significantly Reduced the Occurrence of Rice Blast

**Figure 1:**
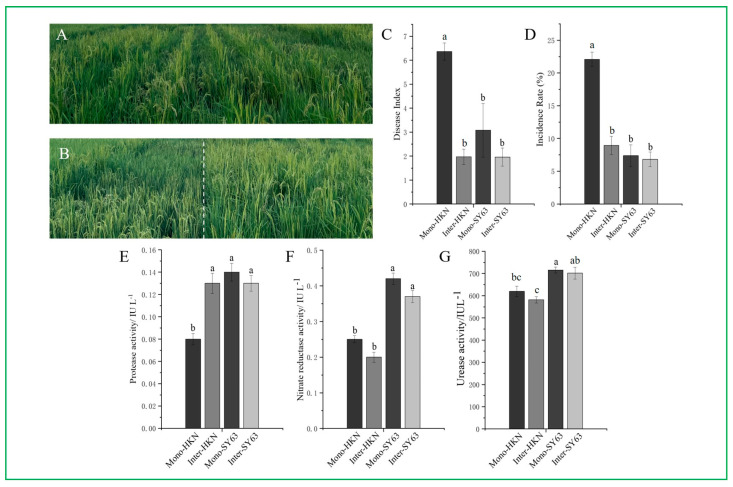
Disease investigation and associated enzyme assay. (**A**) Field image of rice intercropping of Shanyou63 (SY63) and Huangkenuo (HKN). (**B**) Field image of rice monoculture of SY63 and HKN. (**C**) The disease index on rice blast of HKN and SY63 under monoculture and intercropping patterns. (**D**) The incidence rate on rice blast of HKN and SY63 under monoculture and intercropping patterns. (**E**) The protease activity of HKN and SY63 under monoculture and intercropping patterns. (**F**) The nitrate reductase activity of HKN and SY63 under monoculture and intercropping patterns. (**G**) The urease activity of HKN and SY63 under monoculture and intercropping patterns. Mono-HKN indicates rice susceptible variety HKN in the monoculture planting pattern. Inter-HKN indicates rice susceptible variety HKN in the intercropping planting pattern. Mono-SY63 indicates rice-resistant variety SY63 in the monoculture planting pattern. Inter-SY63 indicates rice-resistant variety SY63 in the intercropping planting pattern. Each column represented the average value of twelve independent experiment replicates, and the standard error was represented by the error bars. Letters above the column indicate the significant differences at *p* < 0.05 according to the ANOVA and Tukey’s HSD.

The disease index (DI) and incidence rate (IR) of rice blast were analyzed to elucidate the effect of intercropping on the occurrence of rice blast (Figure 1A,B). Field investigation showed that the panicle blast disease index of Shanyou63 and Huangkenuo were both decreased in the intercropping compared to the monoculture system. The panicle blast occurrence of Huangkenuo in the intercropping (DI_Inter-HKN_ = 1.97, IR_Inter-HKN_ = 8.94%) was significantly decreased than that in the monoculture system (DI_Mono-HKN_ = 6.36, IR_Mono-HKN_ = 22.09%) (*p* < 0.001). The blast disease occurrence of Shanyou63 in the intercropping (DI_Inter-SY63_ = 1.96, IR_Inter-SY63_ = 6.81%) was decreased than that in the monoculture system (DI_Mono-SY63_ = 3.08, IR_Mono-SY63_ = 7.37%), but there was no significant difference (Figure 1C,D). It can be inferred from the above analysis that the implementation of intercropping could effectively reduce the incidence of rice blast, and this approach appears to be more efficacious in reducing the disease severity in the susceptible rice variety Huangkenuo.

### 3.2. Soil Resistance-Related Enzyme Activity in the Rice Intercropping System

Rice rhizosphere soil resistance-related enzyme activities were assayed under intercropping and monoculture systems. There was no significant difference in acid protease, nitrate reductase, or urease activity between Inter-SY63 and Mono-SY63. However, the enzyme activity (acid protease, nitrate reductase, urease) of the resistant variety Shanyou63 was higher than the susceptible variety Huangkenuo. The nitrate reductase and urease activity of HKN in the intercropping (Inter-HKN) had not significantly differed with the enzyme activities in the monoculture (Mono-HKN), but the acid protease activity of HKN in the intercropping (Inter-HKN) significantly increased than that in the monoculture (Mono-HKN) (*p* < 0.01) (Figure 1E–G). In this study, the resistant varieties (SY63) have higher soil enzyme activity than the susceptible varieties (HKN), which was closely related to their stronger disease resistance. This indicated that soil enzyme activities played an important role in rice disease resistance. Huangkenuo, a susceptible cultivar, showed a significant increase in disease resistance after intercropping. At the same time, the activity of soil protease also increased significantly. This suggests that the increase of soil protease activity may be related to the enhancement of rice disease resistance.

### 3.3. Soil Metabolites Detected by LC-MS/MS in Rice Intercropping System

**Figure 2:**
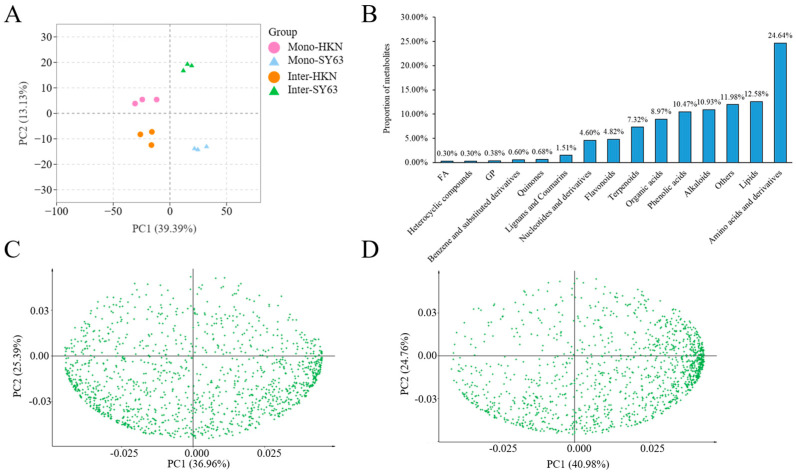
Soil metabolites and principal component analysis. (**A**) PCA of mass spectrometry data of each group of samples and quality control samples. X axis represents the first principal component, and Y axis represents the second principal component. (**B**) Identified metabolite’s types and proportion of two different rice varieties. (**C**) PCA loading plot of soil metabolites of Mono-HKN and Inter-HKN. (**D**) PCA loading plot of soil metabolites of Mono-SY63 and Inter-SY63. Inter-HKN indicates rice susceptible variety HKN in the intercropping planting pattern. Mono-SY63 indicates rice-resistant variety SY63 in the monoculture planting pattern. Inter-SY63 indicates rice-resistant variety SY63 in the intercropping planting pattern.

To screen the metabolites of HKN and SY63 in intercropping and monoculture planting patterns, the UPLC-MS platform was used to detect the primary and secondary metabolites in the rhizosphere soil. Different processing duplicates were found to be clustered together by principal component analysis, demonstrating strong homogeneity and high reproducibility of data between biological replicates (Figure 2A). A total of 15 classes of metabolites were detected, which contained amino acids and their derivatives (24.64%), lipids and lipid molecules (12.58%), alkaloids (10.93%), phenolic acids (10.47%), organic acids and their derivatives (8.97%), and phenylpropanoids and polyketides (5.42%) (Figure 2B). PCA analysis showed that the soil metabolites in Mono-HKN and Inter-HKN, Mono-SY63 and Inter-SY63, were significantly separated along the first principal component (the interpretation rates were 36.95% and 40.99%, respectively). These results indicate that the intercropping pattern significantly affected soil metabolites (Figure 2C,D). The OPLS-DA model well explained the differences between different planting patterns. (R2Y = 1, Q2 > 0.8) indicates that the data of the metabolites’ results were stable and reliable (Supplementary Figure S1).

### 3.4. Differential Metabolites in Rice Intercropping System

**Figure 3:**
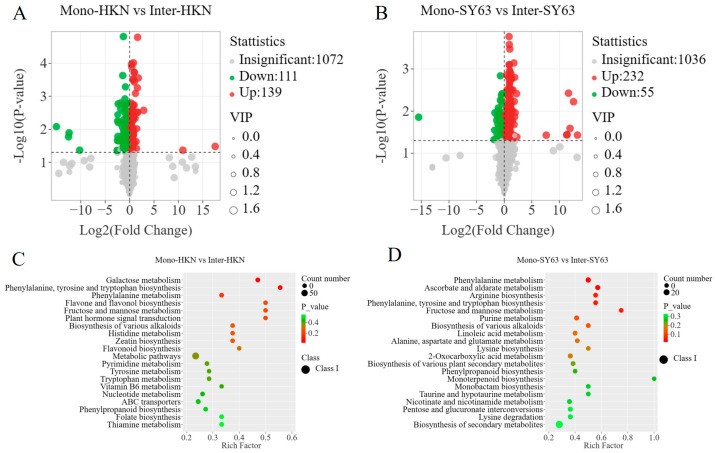
The differential metabolites and metabolic pathways enriched by HKN and SY63 under different planting patterns. (**A**) The volcanic plot of differential metabolites of Mono-HKN and Inter-HKN. (**B**) The volcanic plot of differential metabolites of Mono-SY63 and Inter-SY63. (**C**) The bubble plot of differential metabolic pathways of Mono-HKN and Inter-HKN. (**D**) The bubble plot of differential metabolic pathways of Mono-SY63 and Inter-SY63. Mono-HKN indicates rice susceptible variety HKN in the monoculture planting pattern. Inter-HKN indicates rice susceptible variety HKN in the intercropping planting pattern. Mono-SY63 indicates rice-resistant variety SY63 in the monoculture planting pattern. Inter-SY63 indicates rice-resistant variety SY63 in the intercropping planting pattern.

A total of 205 differential metabolites were screened between Inter-HKN and Mono-HKN; 139 differential metabolites were up-regulated, and 111 were down-regulated (Figure 3A). A total of 287 differential metabolites were detected in the comparison of Inter-SY63 and Mono-SY63, 232 of which were up-regulated and 55 were down-regulated (Figure 3B). It can be seen that there were significant changes in metabolites observed in the disease-resistant varieties (SY63) in the intercropping system. Furthermore, a majority of the significantly altered metabolites in the susceptible variety (HKN) were also originated from SY63. Therefore, it is hypothesized that the enhanced disease resistance in the susceptible variety may be attributed to alterations in metabolites derived from the disease-resistant variety.

### 3.5. The Metabolomic Pathway in Rice Intercropping System Annotated by KEGG

The KEGG database annotated all differential metabolites of different comparison groups in the involved pathways. Enrichment analysis of Huangkenuo (Inter-HKN and Mono-HKN) showed that differential metabolites were significantly enriched in galactose metabolism, phenylalanine, tyrosine and tryptophan biosynthesis, phenylalanine metabolism, flavonoid and flavonol biosynthesis, and fructose and mannose metabolic pathways (Figure 3C). Inter-SY63 and Mono-SY63 treatments illustrated the differential metabolites were significantly enriched in phenylalanine metabolism, ascorbic acid metabolism, arginine biosynthesis, phenylalanine, tyrosine, and tryptophan biosynthesis, fructose and mannose metabolism, purine metabolism, and linoleic acid metabolism pathways (Figure 3D). In these two comparison groups, three metabolic pathways (the phenylalanine metabolic pathway, the phenylalanine, tyrosine, and tryptophan biosynthesis pathway, and the fructose and mannose metabolic pathway) were common pathways. These common pathways synthesize the upstream substances related to plant disease and stress resistance, determining the basis of disease resistance.

### 3.6. Intercropping System Regulates the Assembly of Rhizosphere Bacterial Community

**Figure 4:**
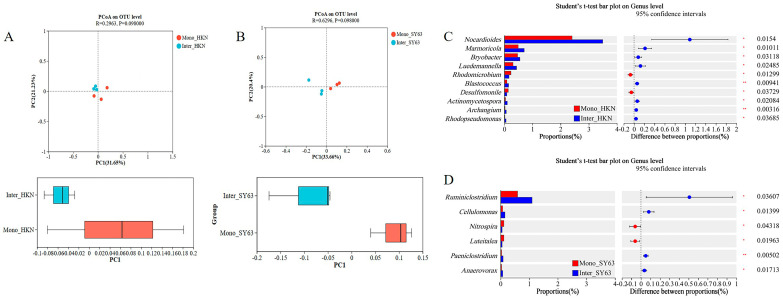
Differential bacterial community of Shanyou63 (SY63) and Huangkenuo (HKN) in intercropping and monoculture. (**A**) Principal component analysis of bacterial communities in HKN under different planting patterns. (**B**) Principal component analysis of bacterial communities in SY63 under different planting patterns. (**C**) Significant differential bacteria genus in Inter-HKN vs. Mono-HKN. (**D**) Significant differential bacteria genus in Inter-SY63 vs. Mono-SY63. Mono-HKN indicates rice susceptible variety HKN in the monoculture planting pattern. Inter-HKN indicates rice susceptible variety HKN in the intercropping planting pattern. Mono-SY63 indicates rice-resistant variety SY63 in the monoculture planting pattern. Inter-SY63 indicates rice-resistant variety SY63 in the intercropping planting pattern.

The 16 S rRNA gene of bacteria was sequenced to examine the influence of two cropping patterns on the community structure of soil bacteria. The results showed that at the class level, the rhizosphere soil bacterial community richness (Sobs index) of Mono-HKN was 146, the Sobs index of Inter-HKN decreased significantly to 138, the Sobs index of Mono-SY63 was 147.33, and the Sobs index of Inter-SY63 decreased significantly to 137.33 (Supplementary Figure S2). PCoA analysis showed that Inter-HKN and Inter-SY63 were separated from the control along PC1, with interpretation rates of 31.65% and 33.66% (Figure 4A,B). This indicated that intercropping changed the composition of the bacterial community in rice soil. The variation of microbial community in resistant varieties Shanyou63 was more significant than that in susceptible varieties Huangkenuo. Bacterial community structure was analyzed and showed that the top ten dominant bacterial phyla were Actinobacteriota, Chloroflexi, Firmicutes, Proteobacteria, Acidobacteriota, Desulfobacterota, Bacteroidota, Myxococcota, Planctomycetota, and Gemmatimonadota (Supplementary Figure S3). Furthermore, 10 bacterial genera were found with clear classification and significant differences in Inter-HKN vs. Mono-HKN, and 6 bacterial genera were found in Inter-SY63 vs. Mono-SY63 in relative abundance (*p* < 0.05), respectively. *Nocardioides*, *Marmoricola*, *Luedemannella*, and *Desulfomonile* were the significant differential bacteria genera in Inter-HKN vs. Mono-HKN (Figure 4C), and *Ruminiclostridium* and *Cellulomonas* were the significant differential bacteria genera in Inter-SY63 vs. Mono-SY63 (Figure 4D). *Nocardioides* were widely distributed in soil and usually decomposed organic matter and released ammonia and other substances, contributing to soil fertility improvement. *Marmoricola* and *Luedemannella* can secrete various enzymes such as protease and amylase, helping to decompose marine organic matter and promote its recycling. In addition, they commonly have bioactivities such as antimicrobials. *Desulfomonile* typically plays an important role in maintaining the ecological balance of water bodies. *Cellulomonas* was usually important in cellulose degradation and environmental protection. These results suggested that in the rice intercropping system, there was a preference for recruiting beneficial bacterial groups that can enhance soil metabolic cycles and disease resistance, thus contributing to decreasing the occurrence of diseases.

### 3.7. Beneficial Microorganisms Were Significantly Related to the Evolved Soil Metabolites

**Figure 5:**
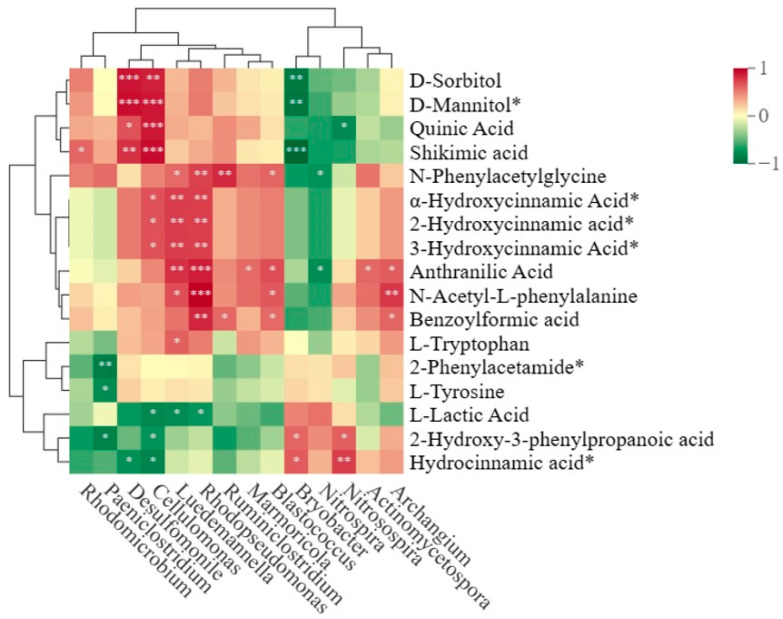
The correlation heat map of soil metabolites and rhizosphere bacterial genus. Asterisk (*) indicates the significance level of the correlation at *p* < 0.05. ** *p* < 0.01, *** *p* < 0.001.

By comparing the KEGG metabolic pathways enriched in different planting patterns between resistant variety ‘Shanyou 63‘ and susceptible variety ‘Huangkenuo’, we found that three metabolic pathways were identified. To further explicate the mutual dependence between significantly changed metabolites and microbes, correlation analysis was conducted between the common differential metabolites of the three metabolic pathways and rhizosphere bacterial communities in rice intercropping systems. D-sorbitol, D-mannitol, quinic acid, and shikimic acid were positively correlated with *Cellulomonas*, which tolerates high pH and catabolizes the major plant cell wall-associated polysaccharides cellulose, pectin, and hemicellulose [34], and *Desulfomonile*, which were regarded as key genera in the intercropping system. Correspondingly, D-sorbitol, D-mannitol, and shikimic acid were significantly negatively correlated with *Bryobacter*, a core genus that plays an important regulatory role in the co-variation network of plant root microbial communities in previous work (Figure 5). These results indicated that D-sorbitol, D-mannitol, quinic acid, and shikimic acid were the core factors reshaping the structure of rhizosphere bacterial communities, thus contributing to the plant disease resistance.

### 3.8. Correlated Metabolites Have Significant Control Effects on Rice Blast

**Figure 6:**
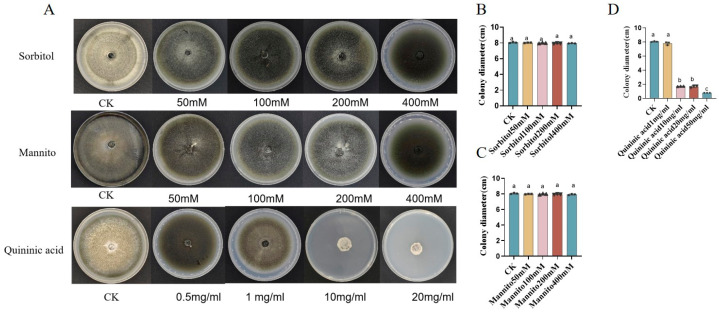
The control effect of metabolites on rice blast of plate-antagonistic assay in vitro. (**A**) The plate-antagonistic graphic of D-sorbitol, D-mannitol, and quinic acid. The text below the plates indicates the corresponding metabolite concentration. (**B**) The pathogen mycelium diameter in the sorbitol-contained PDA plate. (**C**) The pathogen mycelium diameter in the mannitol-contained PDA plate. (**D**) The pathogen mycelium diameter in the quinic-acid-contained PDA plate. Each column represented the average value of three independent experiment replicates, and the standard error was represented by the error bars. Letters above the column indicate the significant differences at *p* < 0.05 according to the ANOVA and Tukey’s HSD.

Three significant correlated metabolites, D-sorbitol, D-mannitol, and quinic acid were selected to confirm the control effect of metabolites on rice blast. The plate antagonistic experiment indicated that D-sorbitol (Figure 6A,B) and D-mannitol (Figure 6A,C) did not exhibit significant inhibitory effects on the mycelium of *M. oryzae*, while quinic acid showed a gradual increase in the inhibition rate with increasing concentration (Figure 6A,D). When the concentration of quinic acid was 50 mg/mL, the inhibition rate achieved 100% (Figure 6D).

**Figure 7:**
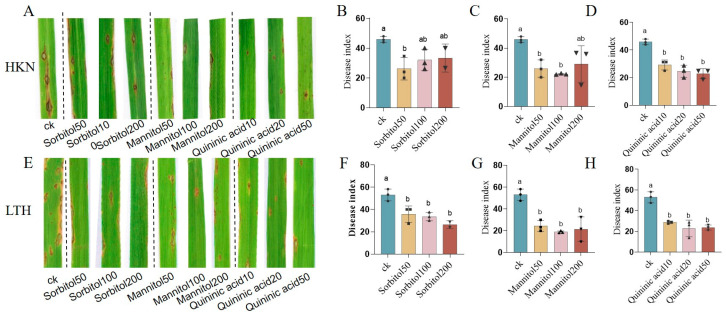
The control effect of metabolites on rice blast of pot experiment in vivo. (**A**) The disease symptoms of HKN after application of D-sorbitol, D-mannitol, and quinic Acid. (**B**) The disease index of HKN after application of D-sorbitol. (**C**) The disease index of HKN after application of D-mannitol. (**D**) The disease index of HKN after application of quinic Acid. (**E**) The disease symptoms of LTH after application of D-sorbitol, D-mannitol, and quinic Acid. (**F**) The disease index of LTH after application of D-sorbitol. (**G**) The disease index of LTH after application of D-mannitol. (**H**) The disease index of LTH after application of quinic acid. Letters above the column indicate the significance among the treatments at *p* < 0.05.

The pot experiment results revealed that, compared to the control, exogenous application of D-sorbitol, D-mannitol, and quinic acid reduced the severity of disease symptoms in the susceptible rice variety HKN and LTH, with smaller lesion areas and fewer lesion scores (Figure 7A,E). Exogenous application of 50 mM D-sorbitol led to a significant decrease in the disease index (Figure 7B), with the control efficacy reaching 42.88%. For another susceptible variety-LTH, the application of D-sorbitol at three concentrations all had significant effects on disease reduction, and the disease index decreased with the increase of concentration (Figure 7F), with the highest disease control effect of 50.11%.

The disease indexes of rice blast of HKN after 50 mM/100 mM/200 mM D-mannitol application were significantly decreased, with the optimal blast-control efficiency being 51.72% when the concentration of D-mannitol was 100 mM (Figure 7C). The application of D-mannitol in three concentrations also had a significant rice blast disease prevention effect on another susceptible variety, LTH (Figure 7G), with the optimal blast-control efficiency being 64.57% when the concentration of D-mannitol was 100 mM.

For quinic acid, the disease index of exogenous application at concentrations of 10 mg/mL, 20 mg/mL, and 50 mg/mL were all significantly decreased, with the increased control efficiency (36.46%, 46.45%, and 50.08%) of accumulated concentrations (Figure 7D). The application of quinic acid in three concentrations also has a significant control effect on rice blast disease of the susceptible variety LTH (Figure 7H), with the optimal blast-control efficiency 56.77. In summary, exogenous application of D-sorbitol, D-mannitol, and quinic acid could enhance the resistance to rice blast, with obviously optimistic control efficacy at concentrations of 50 mM for D-sorbitol, 100 mM for D-mannitol, and 50 mg/mL for quinic acid.

## 4. Discussion

Varieties intercropping are being used with success in many parts of the world, reducing diseases and stabilizing yields [12,35], which provides functional diversity and limits the expansion of pathogens and pests. Our research showed that the resistance of rice varieties to rice blast was increased by the intercropping system, particularly for the susceptible rice variety HKN (*p* < 0.01), highlighting that it can effectively reduce the impact of this fungal disease on crop development as previously demonstrated by Gallet et Raboin et al. [36].

Underground interactions of plant roots improve enzyme activity, promote the absorption of nutrients, and enhance the defense ability against pathogens [37,38]. Since different plant varieties exhibit distinct resource acquisition patterns [39] and vary in their potential to capture solar radiation [40], these variety differences may lead to intervarietal affinity and promote the efficient use of resources. Compared with Mono-HKN, the Acp enzyme activity of Inter-HKN was significantly increased (*p* < 0.01), and NR enzyme and UE enzyme activity were not significantly reduced. There was no significant difference in Acp, NR, and UE enzyme activity between Inter-SY63 and Mono-SY63. These variations could be attributed to litter residues, induced enzyme reactions, and impacts of various rice cultivars in intercropping systems [41]. Overall, these results indicated that intercropping significantly increases soil enzyme activity, and the interaction between rice roots within species enhances disease resistance.

Plant species, genotype, photosynthetic activity, and soil conditions influence the metabolites released by plants into the rhizosphere and bulk soil [42]. Soil metabolites play an important role in plant-plant interaction [28,43], mediating subsurface interactions of intercropping systems through direct or indirect facilitation [23]. In the peanut-maize intercropping system, peanuts modified their root secretion and increased the biosynthesis of flavonoids in the neighboring maize [44,45]. The contents and proportions of amino acids and their derivatives, phenolic acids, and flavonoids were increased by interspecific interactions. Therefore, it can be hypothesized that intercropping alters SY63 soil metabolites in response to adjacent HKN. Root interactions during intercropping alter the bacterial community structure in the rhizosphere of rice in comparison to monoculture [46]. Soil metabolites have a valuable impact on the assembly process of rhizosphere microorganisms [47]. After intercropping with *R. pseudoacacia*, secondary metabolites can inhibit soilborne pathogens, release peanut root secretion, recruit beneficial microorganisms, and regulate bacterial community composition towards positive trends [48]. Neighboring cassava stimulates ethylene release in peanut roots, increases the abundance of Actinobacteria, and reshapes rhizosphere microbial composition [25]. These results suggested that root exudates could be used to assess the adaptations of soil microbial communities to interspecific interactions at the molecular level.

D-sorbitol, D-mannitol, quinic acid, and shikimic acid are important in alleviating plant stress resistance and are positively correlated with *Cellulomonas* and *Desulfomonile*, suggesting that root interaction affects the distribution of rhizosphere bacteria by changing soil metabolites. *Desulfomonile* might be a sensitive indicator of plant response to stress warnings. These indicated that root interaction improved rice blast resistance by changing soil metabolites and rhizosphere bacterial community. The results of this study further indicate that metabolites can be used as signaling molecules to participate in plant defense responses [49]. D-sorbitol, D-mannitol, and quinic acid have been extensively researched in plant disease resistance in recent years. Studies have indicated that sorbitol may enhance the resistance of multiple apple cultivars against ring rot (*Botryosphaeria dothidea*) by activating the salicylic acid (SA) signaling pathway [50]. Mannitol exhibits significant biological functions under stress conditions in organisms. Ming discovered that mannitol enhances the enzymatic activities of catalase (CAT) and superoxide dismutase (SOD) in antagonistic yeast (*Debaryomyces hansenii*), thus strengthening their stress tolerance and ultimately enhancing their efficacy in controlling apple blue mold (*Penicillium expansum*) and gray mold (*Botrytis cinerea*) diseases [51]. Quinic acid, possessing robust antioxidant properties, serves as a natural preservative and antifungal agent. Additionally, as a crucial component of chlorogenic acid, it promotes the synthesis of chlorogenic acid and its derivatives, augmenting the elimination of free radicals and inhibiting lipid peroxidation [52]. In our study, we proved that quinic acid had a significantly inhibitory effect on the mycelium growth of *M. oryzae*, and D-sorbitol, D-mannitol, and quinic acid all had significant blast control effects in vivo.

While our study provides valuable insights into the effects of intercropping on rice blast resistance and the underlying mechanisms, there are several limitations. First, our research was conducted in a specific ecological setting, and the results may not be universally applicable. Future studies should investigate the efficacy of intercropping in different environments and with a broader range of rice varieties. Second, while we identified several key metabolites involved in disease resistance, the complex interplay between these metabolites and the rhizosphere microbial community requires further elucidation. Future research should focus on deciphering the intricate signaling pathways and molecular mechanisms underlying these interactions. Lastly, the long-term sustainability and economic feasibility of intercropping systems need to be assessed to ensure their practical application in agricultural practices.

## 5. Conclusions

Field investigation showed that the panicle blast occurrence of HKN (susceptible variety) in the intercropping was significantly decreased than that in the monoculture system. The protease activity of HKN in the intercropping (Inter-HKN) significantly increased than that in the monoculture (Mono-HKN). Metabolomics analysis showed that the differential metabolites with significant changes are mainly from resistant varieties Shanyou63. KEGG annotation showed that three metabolic pathways (phenylalanine metabolic pathway, phenylalanine, tyrosine, and tryptophan biosynthesis pathway, and fructose and mannose metabolic pathway) were the differential pathways related to rice disease resistance. Microbiome analysis showed that *Nocardioides*, *Marmoricola*, *Luedemannella*, and *Desulfomonile* were the significant differential bacteria genera in Inter-HKN vs. Mono-HKN, and *Ruminiclostridium* and *Cellulomona* were the significant differential bacteria genera in Inter-SY63 vs. Mono-SY63. Correlation analysis indicated that the differential metabolites D-sorbitol, D-mannitol, quinic acid, and shikimic acid were important factors affecting the community structure of *Cellulomona* and *Desulfomonile*, which derived the rice resistance to rice blast. Antagonistic experiments showed that quinic acid had a significantly inhibitory effect on the mycelium growth of *M. oryzae*, and pot experiments showed that D-sorbitol, D-mannitol, quinic acid all had significant blast control effects in vivo.

## Data Availability

The original contributions presented in the study are included in the article/Supplementary Materials, further inquiries can be directed to the corresponding author.

## Supplementary Materials

The following supporting information can be downloaded at: https://www.mdpi.com/article/10.3390/metabo14090507/s1. Figure S1: OPLS-DA models of maize and peanut root exudates under different planting patterns. (A) Mono-HKN vs. Inter-HKN, (B) Mono-SY63 vs. Inter-SY63. Figure S2: Bacterial community richness (Sobs index). Figure S3: Histogram of the relative abundance of bacterial communities.
