# Supplementary material for: Rice Varieties Intercropping Induced Soil Metabolic and Microbial Recruiting to Enhance the Rice Blast (Magnaporthe Oryzae) Resistance
Source: Metabolites. 2024 Sep 20;14(9):507. doi: 10.3390/metabo14090507 (PMC11434330; doi:10.3390/metabo14090507)
Supplement: Supplementary file 1 [file metabolites-14-00507-s001.zip › metabolites-3172537-supplementary.pdf]

Supplementary material

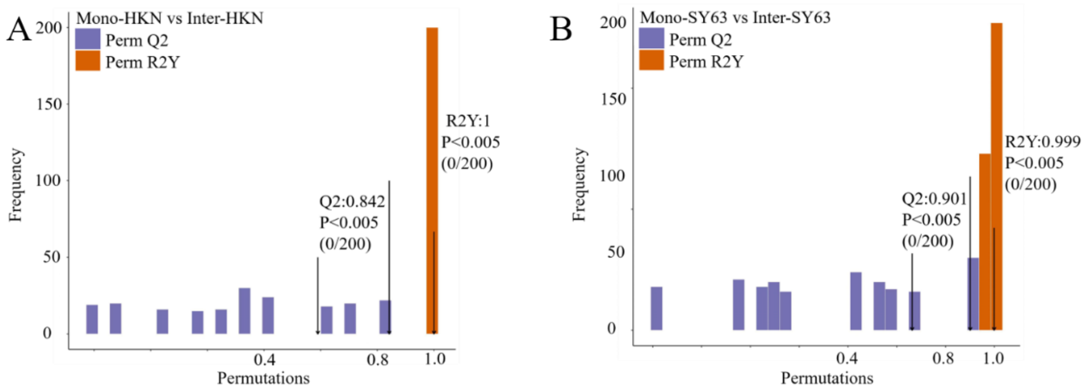

Supplementary Figure S1. OPLS-DA models of maize and peanut root exudates under different planting patterns. (A) Mono-HKN vs. Inter-HKN, (B) Mono-SY63 vs. Inter-SY63. X-axis represents the accuracy of the model, Y-axis represents the frequency of the model classification effect. Mono-HKN indicates rice susceptible variety HKN in the monoculture planting pattern. Inter-HKN indicates rice susceptible variety HKN in the intercropping planting pattern. Mono-SY63 indicates rice resistant variety SY63 in the monoculture planting pattern. Inter-SY63 indicates rice resistant variety SY63 in the intercropping planting pattern.

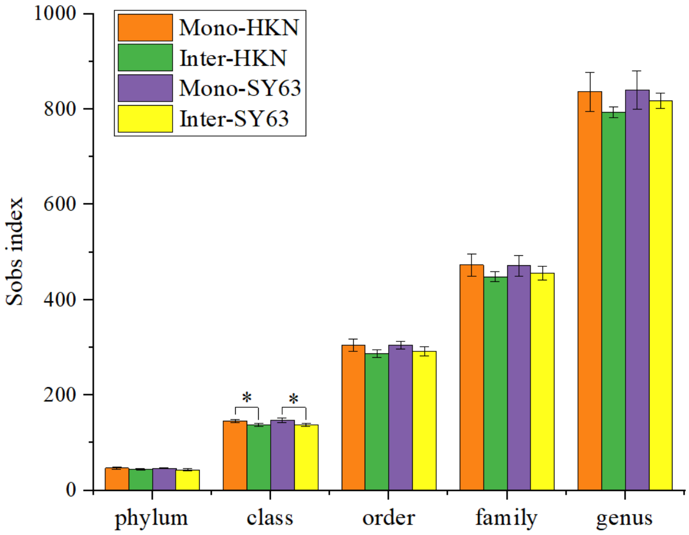

Supplementary Figure S2. Bacterial community richness (Sobs index). Mono-HKN indicates rice susceptible variety HKN in the monoculture planting pattern. Inter-HKN indicates rice susceptible variety HKN in the intercropping planting pattern. Mono-SY63 indicates rice resistant variety SY63 in the monoculture planting pattern. Inter-SY63 indicates rice resistant variety SY63 in the intercropping planting pattern. Each column represented the average value of three independent experiments replicates and the error bars represent standard deviations. Asterisks indicates the significance of differences between the samples. Single asterisk indicates $P < 0.05$, double asterisks indicate $p < 0.01$, triple asterisks indicate $p < 0.001$, ns indicates no significance.

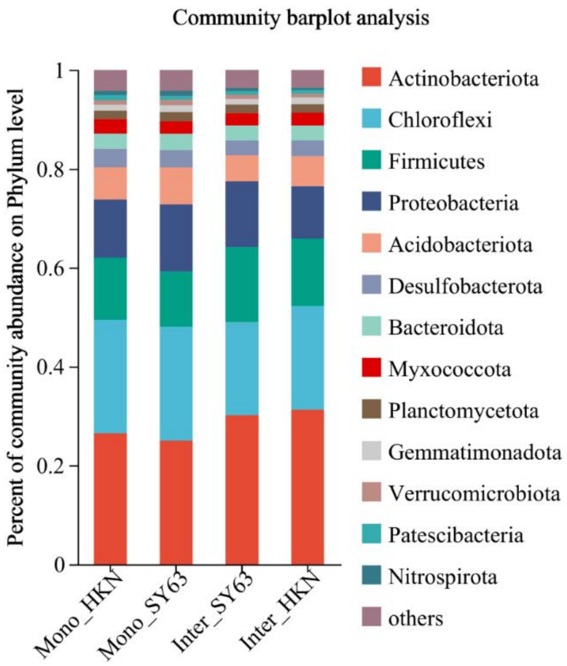

Supplementary Figure S3. Histogram of the relative abundance of bacterial communities. Mono-HKN indicates rice susceptible variety HKN in the monoculture planting pattern. Inter-HKN indicates rice susceptible variety HKN in the intercropping planting pattern. Mono-SY63 indicates rice resistant variety SY63 in the monoculture planting pattern. Inter-SY63 indicates rice resistant variety SY63 in the intercropping planting pattern.
